# Mechanical Strain Stabilizes Reconstituted Collagen Fibrils against Enzymatic Degradation by Mammalian Collagenase Matrix Metalloproteinase 8 (MMP-8)

**DOI:** 10.1371/journal.pone.0012337

**Published:** 2010-08-23

**Authors:** Brendan P. Flynn, Amit P. Bhole, Nima Saeidi, Melody Liles, Charles A. DiMarzio, Jeffrey W. Ruberti

**Affiliations:** 1 Mechanical and Industrial Engineering, Northeastern University, Boston, Massachusetts, United States of America; 2 Electrical and Computer Engineering, Northeastern University, Boston, Massachusetts, United States of America; 3 Center for Engineering and Medicine, Massachusetts General Hospital, Harvard Medical School, Boston, Massachusetts, United States of America; 4 School of Optometry and Vision Sciences, Cardiff University, Cardiff, United Kingdom; Illinois Institute of Technology, United States of America

## Abstract

**Background:**

Collagen, a triple-helical, self-organizing protein, is the predominant structural protein in mammals. It is found in bone, ligament, tendon, cartilage, intervertebral disc, skin, blood vessel, and cornea. We have recently postulated that fibrillar collagens (and their complementary enzymes) comprise the basis of a smart structural system which appears to support the retention of molecules in fibrils which are under tensile mechanical strain. The theory suggests that the mechanisms which drive the preferential accumulation of collagen in loaded tissue operate at the molecular level and are not solely cell-driven. The concept reduces control of matrix morphology to an interaction between molecules and the most relevant, physical, and persistent signal: mechanical strain.

**Methodology/Principal Findings:**

The investigation was carried out in an environmentally-controlled microbioreactor in which reconstituted type I collagen micronetworks were gently strained between micropipettes. The strained micronetworks were exposed to active matrix metalloproteinase 8 (MMP-8) and relative degradation rates for loaded and unloaded fibrils were tracked simultaneously using label-free differential interference contrast (DIC) imaging. It was found that applied tensile mechanical strain significantly increased degradation time of loaded fibrils compared to unloaded, paired controls. In many cases, strained fibrils were detectable long after unstrained fibrils were degraded.

**Conclusions/Significance:**

In this investigation we demonstrate for the first time that applied mechanical strain preferentially preserves collagen fibrils in the presence of a physiologically-important mammalian enzyme: MMP-8. These results have the potential to contribute to our understanding of many collagen matrix phenomena including development, adaptation, remodeling and disease. Additionally, tissue engineering could benefit from the ability to sculpt desired structures from physiologically compatible and mutable collagen.

## Introduction

In vertebrates, fibril-forming collagens (types I, II, III, V and XI), a highly-conserved family of self-assembling triple helical molecules, are the predominant tensile load-bearing proteins in the connective tissue of the musculoskeletal system (bone, ligament, tendon, cartilage, intervertebral disk) and in more specialized tissues such as the corneal stroma, blood vessels and skin. Fibrillar collagens are generally organized and packed into structures which appear to epigenetically adapt to their mechanical environment. From development through adulthood in a healthy animal, collagen monomers are constantly being synthesized and degraded [1]. Despite this continual turnover, connective tissue generally remains well-adapted such that the load-bearing fibrils persist in the path of the applied mechanical strain (even during growth).

Research suggests that control of epigenetic adaptation of connective tissue ECMs is largely provided by resident fibroblasts which transduce mechanical loads to effect an optimized remodelling response [2]–[9]. The specific manner by which cells sense and then convert mechanical forces into signaling events is the subject of intense research [10]–[14]. In a recent investigation, Del Rio *et al.* [15], demonstrated that binding affinity of vinculin to talin (a membrane integrin complex) is strain-controlled. Such strain-sensitive mechanisms may produce fibroblast responses to mechanical stimuli which include changes in differentiation state [16], orientation/morphology [17]–[19], proliferation [20], migration patterns [19], [21]–[23] and in protein and cytokine expression including expression of collagens, matrix metalloproteinases (MMPs) and tissue inhibitors of matrix metalloproteases (TIMPs) [16], [19], [24]–[32]. Examination of fibroblast behavior in general suggests that they interact with the ECM through binding of matrix fibrils (via focal adhesions) [33], [34], cytoskeleton-generated traction forces [35], [36] and the secretion of ECM catabolic and anabolic molecules. During adaptive matrix remodeling under load, mechanically-activated fibroblasts secrete both synthetic and degradative ECM molecules [25], [27]–[29]. Though it is clear that epigenetic adaptation and growth of collagen-based load-bearing matrix requires some form of preferential placement and removal of collagen, there has been no specific mechanism identified by which fibroblasts can simultaneously remove unloaded collagen fibrils while reinforcing loaded ones.

Since the initial discovery of native interstitial collagenases in the resorbing amphibian tail by Gross in 1967 [37] significant questions have remained regarding the mechanisms which control the indiscriminate cleavage of collagen. In vertebrate animals, fibroblasts utilize a limited set of enzymes to degrade the native collagen triple helix. The two major families of collagen cleaving enzymes (MMPs and cathepsins) are actively involved in matrix formation, remodeling and homeostasis [37]–[39]. For comprehensive review of MMP function and activity see [40]. Some members of the zinc-dependent MMP family of collagenases (such as MMP-1 and MMP-8) cleave the native type I collagen triple helix preferentially at a highly-conserved site located between Gly_775_ and Ile_776_. It has been shown that mutations at or around this site render the collagen resistant to MMP collagenases *in vitro* [41]. When similar mutations are introduced into a murine model, mice develop a generally fibrotic phenotype indicating that *selective* collagen turnover is necessary for proper maintenance of matrix [42]. Part of the answer is provided by secreted regulatory molecules (e.g. TIMPs) which inhibit enzyme activity and help refine the balance, deposition, and destruction of collagen (for review see [43]). However, the simple release of enzymes, collagen, and inhibitor will not lead to organized remodeling.

Recently, we proposed a mechanochemical model to explain the formation and maintenance of load-bearing connective tissue [44], [45]. The model requires: 1) spontaneous self-assembly of collagen into highly-organized arrays (ample evidence[46]), 2) selective removal of unloaded collagen and 3) preferential incorporation of collagen into loaded fibrils. The latter two conditions have yet to be clearly demonstrated under conditions similar to those found *in vivo*. There is a significant body of research suggesting that collagen is stabilized by mechanical strain against both thermal denaturation [47] and enzymatic degradation [44], [45], [48]–[50]. We have developed and tested an environmentally-controlled, loading microbioreactor compatible with high-resolution optical microscopy and label-free image processing algorithms [44]. Here we show, for the first time, that mechanical strain enhances the persistence of reconstituted collagen fibrils in the presence of a highly specific, physiologically relevant, collagen cleaving enzyme: MMP-8.

## Results

### Qualitative visual observation of the differential interference contrast (DIC) images suggests that collagen fibrils persist longer in the presence of MMP-8 when strained

**Figure 1:**
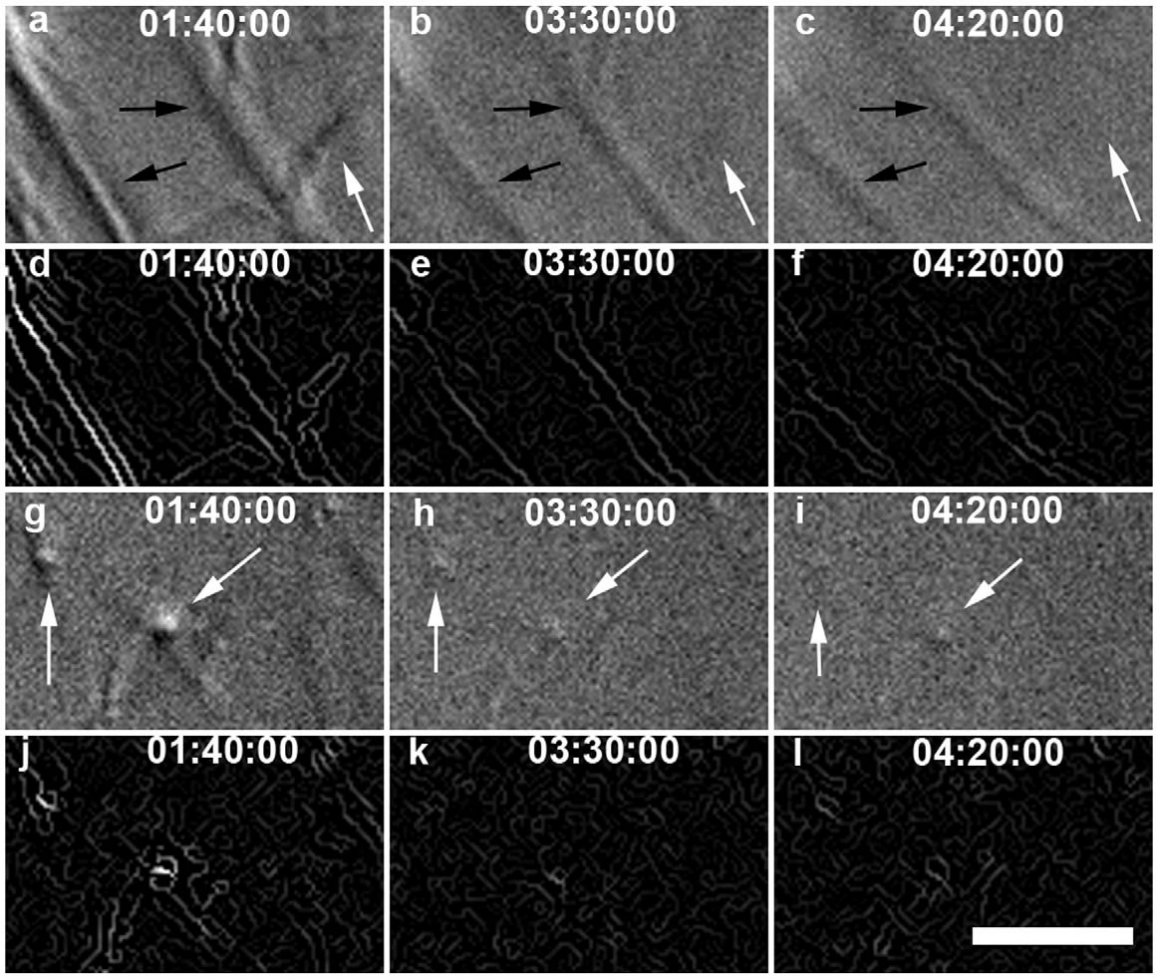
DIC and Edge detection results for loaded ROI #8 (A–F) and unloaded ROI #4 (G–L) from Fig. 5. (A–C, G–I) DIC image series of degradation, with intensity adjusted. (D–F, J–L) Edge detected intensity image series of degradation. In strained ROI#8, the direction of strain, and the orientation of the predominant strained fibrils, is northwest-southeast (compare with Fig. 5). Black arrows denote strained fibrils, white arrows denote unstrained fibrils. Note how quickly the perpendicular, unstrained fibrils (seen as high contrast dots) fade compared to strained fibrils. Also note relatively unstrained fibrils in the strained ROI degrade much faster than strained fibrils. Bar  = 5 µm. (see SI movie for video of this sequence and accompanying text).

**Figure 2:**
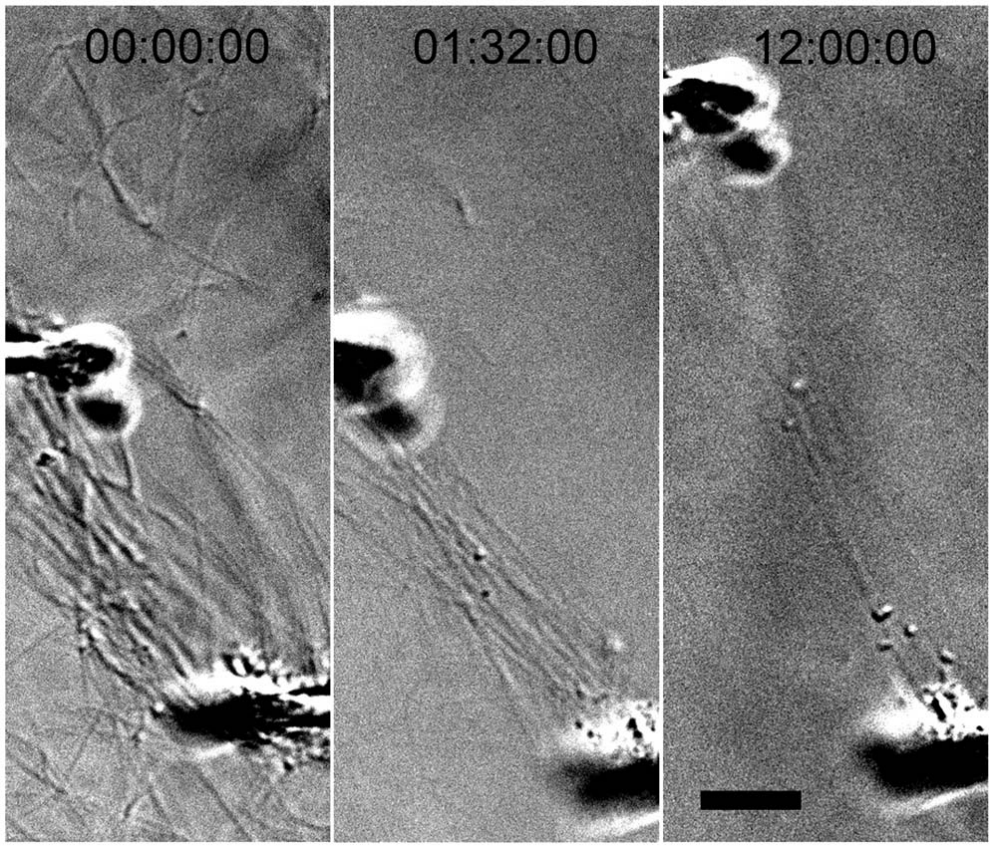
DIC image showing pipette drift stretching fibrils to over 100% initial length during enzymatic degradation. Experimental time series images show the micropipettes drifting 30–40 µm apart, without fracturing or significantly thinning attached collagen fibrils, indicating either additional collagen deposition or structural changes within the fibrils. Note, in the last frame, stretched fibrils remain visible in the microbioreactor while peripheral fibrils have degraded. Experiments with large pipette drift were excluded from analysis. Bar  = 10 µm.

From direct visual assessment of collagen micronetwork behavior it was clear that the strained region is more dense than the peripheral fibril network prior to the degradation process (n = 8). In addition, it could be readily observed that one or more strained fibrils remain between pipettes well after all peripheral fibrils have degraded (n = 8). In the unloaded networks, large fibrils perpendicular to the field of view (and the direction of stretch) can often be observed as high contrast dots with light and dark edges (DIC effect). These dots are easy to visually track throughout the degradation event and cannot move out of the thin DIC focal plane because they are perpendicular to it. These perpendicular fibrils appear to lose intensity more quickly than the fibrils oriented in between pipettes (Fig. 1). In some experiments (n = 3, excluded from analysis) one or both of the pipettes drifted far from the starting position, greatly increasing the separation distance and fibril stretch (∼100% increase). Despite this drift, one or more of the strained fibrils persisted between the pipettes after peripheral unstrained fibrils had degraded (Fig. 2).

### Quantitative analysis of the rate of fibril degradation confirms visual assessment that strain enhances collagen fibril survivability

**Figure 3:**
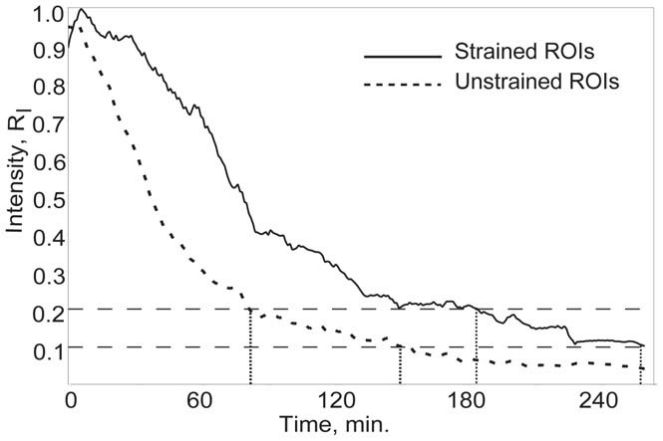
Normalized edge intensity vs. time (minutes) for loaded (solid line) and unloaded (dashed line) ROIs shown in fig. 5. Typically, the noise threshold of the edge intensity algorithm is somewhere between R_I_ [0.1, 0.2]. Note the large differences in degradation time between loaded and unloaded ROIs when R_I_  = 0.1 or R_I_  = 0.2 are used as end points (110 or 120 min. respectively).

**Figure 4:**
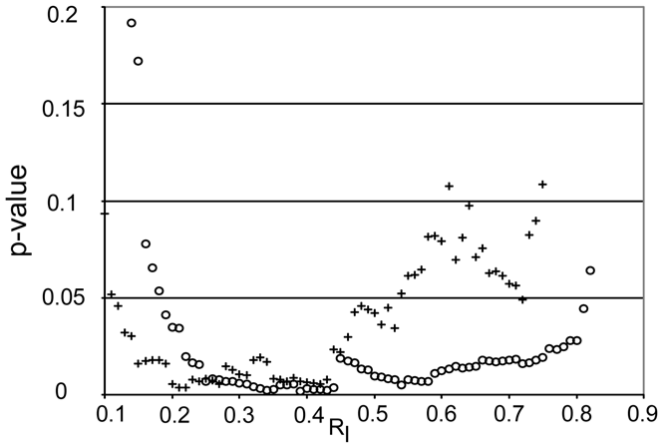
P-value for degradation end time vs. R_I_ for total edge intensity (o) and directional edge intensity (+). Note the differences between total and directional edge intensity methods. While both methods show significance (p<0.05) over a large range of R_I_, the total edge intensity is statistically different over almost the entire regime [.19, .81], while directional edge intensity is statistically different over the range from [.12, .53].

A conservative quantitative edge detection algorithm (described in detail in Bhole *et al* [44]) was used to estimate the rate of degradation of the fibrils in the microbioreactor. Fig. 1 shows a sequence of images depicting regions of interest (ROIs) located in the strained portion of the collagen network (1A–C DIC image; 1D–F edge detected image) and in the unstrained portion (1G–I DIC image; 1J–L edge-detected image). The edge-detected, processed images were integrated to produce time varying edge intensity values reflecting the total quantity of fibrillar collagen, weighted to largest fibrils, in the ROI at any time (Fig. 3). The time required to degrade the observed fibrils to a fraction, R_I_, of the initial integrated DIC edge intensity varied significantly (p<0.05) between strained fibrils and unstrained control fibrils over R_I_ [0.19, 0.81] for total edge intensity and over R_I_ [0.12, 0.52] for directional edge intensity (Fig. 4). The data extracted from the quantitative analysis of separate unstrained and strained ROIs clearly indicates a faster degradation rate for the unstrained network as determined by both total and directional edge intensity methods. Degradation time (defined as R_I_  = 0.2) was 9822±3648 s (mean±std) (strained) and 7805±2321 s (unstrained) for directional edge intensity, and 9076±2769 s (strained) and 7467±1738 s (unstrained) for total edge intensity.

## Discussion

To perform this investigation, we first had to develop and test a label-free, environmentally-controlled, mechanochemical, micro (to mitigate the cost of MMP) collagen degradation assay. The results of this effort were published using a robust but physiologically less relevant (and cheaper) bacterial collagenase (BC) as the degrading enzyme[44]. By combining DIC optical microscopy with custom image processing, degrading collagen fibrils in a micronetwork could be tracked to a theoretical lower diameter limit of ∼50 nm, with dynamic resolution of Δ10 nm, minimizing the need for potentially kinetics-altering labeling of the enzymes or the collagen[44]. In the bioreactor, reconstituted type I collagen micronetworks are readily mechanically manipulated by functionalized micropipette tips to produce a tensile strain field. Importantly, the experimental design provides internal, paired control fibrils for each experiment (fibrils not strained between the pipettes), which removes complications resulting from variability in the preparation.

In this investigation, we have found that strained, reconstituted collagen fibrils persist significantly longer in the presence of MMP-8 than paired, unstrained control fibrils. The result was visually obvious as fibrils between the pipettes often remained visible long after unloaded fibrils were impossible to observe. Quantitatively, the difference in the degradation rate detected with our custom edge detection and image processing algorithm corroborated the visual data. We suggest that the enhanced persistence of the strained collagen fibrillar aggregates is the result of a strain-induced reduction in enzymatic cleavage rate. However, due to limitations of our system (no cross-links, no telopeptides), we were not able to discern the details of what we expect is a sensitive and continuous relationship between strain and fibril retention. This is because the *actual* strain on fibrils in the micronetwork cannot be precisely controlled or measured due to the possibility of intrafibrillar sliding of monomer. Though sliding could reduce the applied strain or alter molecular packing within fibrils, these potential effects make the observations conservative and the overall effect is fibril stabilization against enzymatic cleavage.

### Reconstituted atelocollagen vs. native collagen

#### Telopeptides

It must be noted that the pepsin-extracted collagen used in this study has very few telopeptides (non-helical ends of native collagen molecules). Fibrillogenesis time increases and molecular packing order decreases with removal of telopeptides, though characteristic 67 nm banding remains [51]. While stability against thermal and collagenolytic degradation by bacterial collagenase have been shown to be similar for collagen fibril films reconstituted from pepsin extracted and acetic acid extracted (telopeptides intact) type I bovine collagen, the presence of the C-terminal telopeptide has been shown to alter MMP kinetics by reducing accessibility [52], [53].

#### Thermal unfolding

While type I collagen molecules have been shown to thermally unfold at physiological temperature, the timescale is much longer (∼10 hours) than the experiments performed here [54].

#### Lack of crosslinks

Partial inhibition of cross-linking has been shown to significantly reduce tissue stiffness in vivo [55] and complete lack of cross-links puts an upper limit on the mechanical force which can be imparted as the fibrils are principally held together by intermolecular hydrogen bonds. However, uncross-linked reconstituted collagen gels have been characterized as non-linear viscoelastic [56] with elastic moduli of 0.2MPa at low strain rates at 6% strain [57] (modulus increases with applied strain, 6% was the highest tested in the study). Similar uncross-linked gels have been used, with viscoelastic properties taken into account, to measure cell traction forces [58]. Additionally, stable cross-links form slowly in vivo from labile intermediates, on the timescale of weeks [59].

#### Molecular packing

It has been shown that the packing of collagen monomers into insoluble fibrils increases their resistance to collagenolysis due to steric hindrance [53]. The lack of telopeptides, and resulting decrease in packing order, should increase susceptibility of fibrils to collagenolysis. Additionally, possible molecular sliding induced by the application of strain could further expose individual molecules, making the results conservative. It is unlikely that reduced fibrillar packing order decreases the MMP susceptibility of collagen fibrils. Relevance: Though intact telopeptides reduce the collagenolytic rate of MMP, the strain-protection phenomenon is seen in native tissues as well [60], [61], which indicates the results seen here are not due entirely to lack of intact telopeptides. The higher collagenolytic rate does not preclude the qualitative findings of strain-protection because these results are based on relative rates of degradation. *in vivo*: These results may not directly relate to collagen turnover in older, stiffer, more heavily cross-linked fibrils, though the mechanism may be similar. The uncross-linked fibrils we analyze here may be representative of newly-synthesized, native, collagenous networks before significant cross-linking. Given the myriad of different load magnitudes which are both temporally and spatially varying *in vivo*, we would expect the mechanochemical relationship between collagen and MMP to be fairly sensitive at low loads and strains. That way, collagen under marginal tension (e.g. skin) would be preferentially retained over completely unloaded collagen. *in vitro*: The mechanochemical relationship between enzymatic cleavage and applied strain shown here could be exploited, by controlling the network strain state and introducing enzymes, to produce highly organized tissue engineering constructs which can be actively remodeled in vivo.

### Unstrained fibrils within the strained ROI mask actual effect of strain on degradation rate, but provide additional control data

It should be noted that, within the strained micronetwork, there must be some fibrils which do not carry significant tensile load. This is a necessary consequence of stretching a network uniaxially [62]. An example of unstrained fibrils oriented at nearly right angles to the applied load can be observed in the first panel of Fig. 1 (A). Such unstrained fibrils in the “strained” ROI are necessarily included in the quantitative analysis and generally make both the data and subsequent statistical analysis more conservative. Without these unstrained fibrils, the strained ROIs would display an even lower collagen cleavage rate. However, because these structures are so close to the strained fibrils, they provide excellent additional and tightly-paired control data. In the sequence shown in Fig. 1 (A–C) and in the supporting information Video S1, one can see the MMP is preferentially removing unstrained material from around the strained fibrils.

### The effect of density differences for strained vs unstrained fibrils

Networks were typically denser in the inter-pipette area. The mean ratio in integrated edge intensity between loaded and unloaded ROIs was 2.87 (max 7.04, SD. 1.80) for total edge intensity images and 2.30 (max 3.05, SD. 0.64) for directional edge intensity images. This is principally due to matrix compaction perpendicular to the direction of strain. However, there is also the possibility that mechanical strain enhances polymerization of free monomer into loaded fibrils. We have noted this effect previously (Bhole *et al* [44]) and during the course of this investigation, and it requires further investigation.

#### Diffusion

MMP-8 has a molecular weight similar to that of Bovine Serum Albumin [63], which has been well characterized (Stokes' radius 3 nm, diffusion coefficient 6e-7 cm^2^/s [64]) and is thus assumed to have similar diffusive properties. Gels are sparse, ∼0.0005% V/V solution before addition of enzyme, so the free solution diffusion coefficient is used. The denser region of fibrils between the pipettes has a width of 15±5 µm, with a maximum diffusion time L^2^/D of ∼1.6 s. A 10x decrease (conservative estimate) in local diffusion coefficient due to increased fibril density should have a near negligible, ∼16 s, effect on the overall enzyme gradient and degradation rate compared to the experiment timescale (2–4 hours). The diffusion time estimates are an upper bound, for we are considering a cylindrical region with enzyme available on all sides, not a 1-dimensional slab.

#### Reaction-diffusion enzymology on denser fibril array

With a sparse fibrillar gel and a low concentration of enzyme, the time-dependent ratio of remaining un-degraded collagen to initial collagen, though dependent on enzyme concentration, is independent of initial collagen concentration, assuming similar sized fibrils ([65], eq. 112). Therefore, comparison of normalized edge intensity between ROIs with different starting fibril densities, but similar sized individual fibrils, provides an accurate comparison of degradation rates.

### Can strain-mediated collagen stability shed light on the progression of degenerative collagen-related diseases in compressive soft-tissue?

In humans, with increasing age, the balance of collagen removal and replacement is lost with deleterious effects [43]. Such diseases include devastating conditions such as osteoarthritis (OA) and degenerative intervertebral disk disease which have an enormous impact on the quality of life of the individual. In compressively loaded soft tissue such as cartilage and intervertebral disk (IVD) collagen is kept in tension through the swelling pressure imparted by the resident proteoglycans (PGs) [66], [67]. For IVD degeneration, the degradation of collagen in the anulus fibrosus might be explained in part by the loss of stabilizing tension. Normally, the nucleus swelling pressure (which is generated by PGs) puts the anulus collagen fibrils in a state of relative tension. With age, both the PG content and swelling pressure decrease [67], reducing the disk vertical space and the tension on the anulus collagen fibrils. By the strain-stabilization hypothesis, loss of tension may reduce the collagen fibril resistance to degradation. This possibility was suggested in 2004 [45] and is supported in a recent experiment in which tension applied to anulus fibrosus was shown to protect collagen structure in an *in vivo* rat model of a disk degenerating injury [60]. These *in vivo* findings may indicate a potential *in vivo* correlation for *in vitro* strain-stabilization findings. In OA, the principal manifestation of the disease is a progressive loss of the type II collagen network, presumably through enzymatic degradation [68]. In normal cartilage, the relatively high concentration of fixed negative charges on trapped PGs places the restraining collagen fibrils under significant tension [66]. PG loss (with age) could significantly reduce protective tension in the collagen network, thus making fibrils more susceptible to enzymatic collagenolysis. Loss of collagen network integrity can lead to escape of trapped PGs and further loss of collagen tension. Though OA involves mainly type II collagen, several enzymes from the MMP family, including MMP1, MMP8 and MMP13, hydrolyze collagen types I-III via similar mechanisms (for review see [69]) and the strain-stabilization mechanism could be shared across structural fibril forming collagens. For both OA and IVD degeneration, the concept of strain-stabilization of collagen against degradation, with few other considerations (e.g. loss of PGs), could be adequate to explain progression of the disease. It is interesting to note that in both IVD degeneration and OA, the earliest detectable event appears to be loss of PG induced swelling pressure. We suggest that in these compressive tissues, PG loss relieves resident collagen fibrils of their protective tensile strain.

### Can preferential retention of loaded collagen explain directional remodeling and load optimization?

There is substantial evidence that, during growth and remodeling of extra-cellular matrix, the presence of both MMPs and fibrillar collagens is necessary to produce a “load-adapted” structure [37]–[39], [42]. It is well-known that fibroblastic cells possess contractile machinery which applies local tensile strains to the extra-cellular matrix [35], [70]. These strains may affect the rate of enzymatic cleavage of fibrils, the rate of monomer incorporation into fibrils, or both. If the cleavage rate of fibrillar collagen by MMPs and the rate of monomer incorporation into fibrils are even modestly affected by tensile mechanical strains, there are significant implications for new insights into epigenetic, load-adaptive connective tissue development and remodelling. Specifically, the strain state of the matrix would be sufficient to determine the susceptibility of individual collagen fibrils to degradative enzymes secreted by activated fibroblasts. Fibril incorporation of newly secreted collagen intermediate filaments or monomers, if dependent on fibril strain, could be predicted or controlled via the same matrix strain state. The potential effect of strain on the rate of monomer incorporation into fibrils during fibrillogenesis requires further investigation.

### Conclusion

Given the potential of strain-stabilization of collagen to explain so many phenomena in structural biology, the mechanochemical relationship between strain, collagen monomers and MMPs merits further and more detailed exploration. The observed strain-protection of collagen fibrils has significant potential to contribute to our understanding of numerous phenomena which are observed in vertebrate structural biology and to open new routes in tissue engineering. Additionally, as was previously shown with bacterial collagenase [44], application of strain and exposure to collagenolytic enzyme leads to preferential remodeling *in vitro* of collagen networks. This finding has implications for tissue engineering and should be investigated further to characterize the mechano-chemical relationship. The susceptibility of native collagen fibrils to MMP cleavage should be tested for different levels of applied strains and stresses, both static and cyclic.

## Materials and Methods

### Experimental Design

The experimental setup and equipment were as described in Bhole et al [44]. The bioreactor used was a custom, 3 mm diameter, 40 µL working volume, insert glued to a Delta T Culture Dish with #1.5 cover glass, with Culture Dish Controller and Objective Heater for thermal control (Bioptechs, Butler, PA). Micropipettes were created in a micropipette puller (#P-97, Sutter Instrument, Novato, CA), tip size adjusted to 10±5 µm by breaking against a glass cover slip, plasma cleaned (#PDC-32G, Harrick Plasma, Ithaca, NY), functionalized with 3-Mercaptopropyl-trimethoxysilane (63800, Sigma-Aldrich, St. Louis, MO) and GMBS (22324, Thermo Scientific, Waltham, MA), and positioned, using two Eppendorf Transferman NK2 micromanipulators, ∼50 µm above the glass and 5–10 µm apart. Bovine type I collagen (#5005-B, Advanced Biomatrix, San Diego, CA) was reconstituted in the bioreactor at 37°C, 2.7 mg/ml in Tris-HCl Buffer (pH 7.4, 50 mM Trizma, 0.2 M NaCl, 5 mM CaCl_2_), under non-drying immersion oil (Type A, Cargille, Cedar Grove, NJ) to prevent evaporation. Fibrils precipitated around functionalized micropipettes for at least 1 hour prior to addition of enzyme. Pipettes were moved 10–20 µm apart immediately prior to addition of enzyme. Recombinant Human MMP-8 (#5001, Chondrex, Redmond, WA) was thawed, activated with 1 mM APMA, separated into 10 µL, 1.5 µM, vials, and immediately refrozen at −80C until use. Prior to use, activated MMP-8 was incubated at 37°C for 1 hour. Differential interference contrast (DIC) images were taken throughout gelation and degradation using a TE2000E (Nikon, Japan) inverted microscope with a 60x, 1.45NA Plan Apochromat objective and a digital camera (CoolSNAPHQ2 1394, Photometric, Pleasanton, CA).N = 13 prior to image processing screening.

### Image and Data Analysis

**Figure 5:**
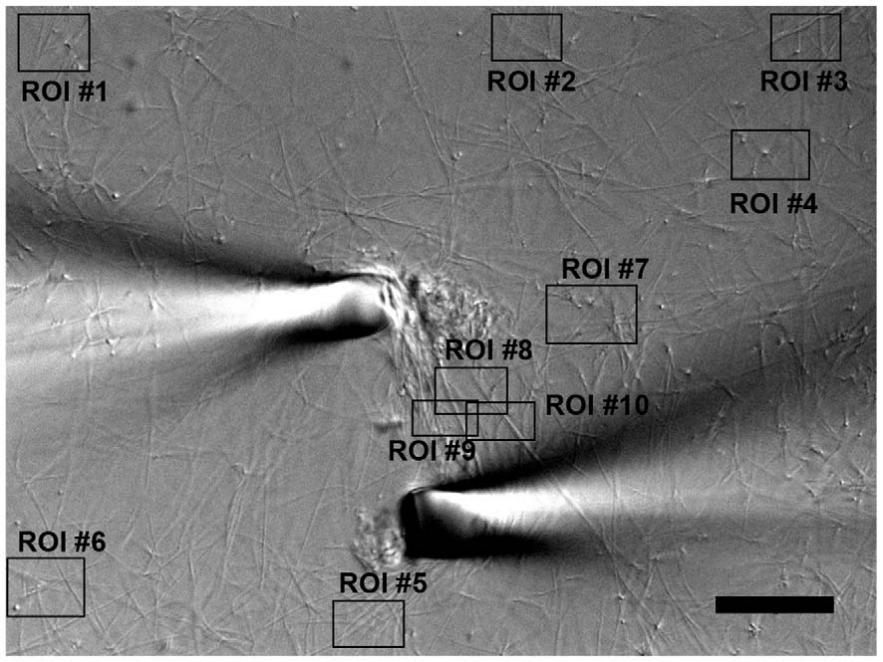
Experimental setup and ROI selection. Each experiment began with collagen polymerized in a bioreactor, both between functionalized pipettes and in the periphery. For edge intensity integration, ROIs were selected, using the same general pattern each time, but choosing ideal locations in each section to monitor large perpendicular or parallel fibrils. Note the large perpendicular fibrils in ROI #4. These fibrils make an excellent visual control, for while they provide little weighted edge intensity, they are easy to track visually and are ideal control fibrils – they should experience little or no strain. Bar  = 20 µm.

Processing and analysis were performed in MATLAB (The MathWorks) similar to the methods of Bhole et al [44]. For each experiment, 10 ROIs were selected from fibrils stretched between pipettes (3–4) or fibrils on the periphery, not stretched between pipettes (6–7), see Fig. 5. A standard pattern was followed except when pipette placement or minor drift excluded certain ROIs. Experiments with large pipette drift (Fig. 2) were excluded from analysis. For each ROI series, the intensity was adjusted based on a window (mean, left width, right width) normalized to the first 5 frames of degradation. Edges were detected using a customized Canny-Deriche algorithm that output a grayscale (8-bit, [0, 255]) edge image as well as 4 grayscale directional edge images containing all prominent edges in a given 45° cone (North-South, EW, NWSE, or NESW). To account for noise, a noise threshold, I_N_, was defined for each ROI series as the intensity value higher than 99% of edge image intensity values averaged over five void images (end of degradation, section of ROI with no fibrils). Edge intensity for each image was organized via histogram ([I_N_:255],[counts]) and integrated via dot product ([I_N_:255]•[Counts]^T^), i.e. weighted summation, emphasizing largest relative edges (fibrils) in each ROI, and ignoring values below noise threshold I_N_. Output was five time series of integrated edge intensity, one total and four directional. Each ROI series was examined frame by frame for detrimental anomalies such as bubbles (high edge intensity), fibril drift (out of ROI or out of focal plane), and pipette drift, and any corrupted ROIs were omitted from further data analysis. After strict examination, N = 8 experiments remained useable. For each experiment the total edge intensity series as well as the directional edge intensity series most closely aligned with direction of stretch were used. Edge data was processed by correcting any all-ROI intensity jumps via a proportional multiplier (applied to all ROIs in an experiment), smoothing data, averaging data in two groups of ROIs (loaded, unloaded), and normalizing each group to [0,1]. End time, time to reach a fraction, R_I_, of initial edge intensity, was calculated. End time was compared between stretched and unstretched ROI groups, for both total and directional edge intensity, using paired student's t-tests on the range R_I_ [0.1, 0.9]. Though the samples sizes were statistically small, distributions were within 2 standard deviations and normal probability plots were linear. See supporting information File S1 for a more complete discussion on the affects of DIC bias, differences in fibril density between strained and unstrained RIOs, and diffusion delays.

## Supporting Information

File S1 Additional information on finer aspects of methods.
(0.05 MB DOC)

Video S1 Degradation series contrasting strained and unstrained ROIs. Top Left) Entire field of view showing a collagen network, reconstituted around functionalized micropipettes, degrading in the presence of MMP-8. Bar  = 10 µm. Top Right) ROI #4 - unstrained fibrils, including two perpendicular fibrils. Bar  = 1 µm. Bottom Left) ROI #8 - strained fibrils. Note some fibrils within the strained ROI are perpendicular to the strain direction (Black Arrow), and these fibrils disappear before the strained fibrils. Bar  = 1 µm. Bottom Right) Normalized, integrated edge intensity from ROI #4 (unstrained) and ROI#8 (strained) versus time.
(2.09 MB AVI)
