# Supplementary material for: Mechanical Strain Stabilizes Reconstituted Collagen Fibrils against Enzymatic Degradation by Mammalian Collagenase Matrix Metalloproteinase 8 (MMP-8)
Source: PLoS One. 2010 Aug 23;5(8):e12337. doi: 10.1371/journal.pone.0012337 (PMC2925882; doi:10.1371/journal.pone.0012337)
Supplement: File S1 — Additional information on finer aspects of methods. (0.05 MB DOC) [file pone.0012337.s001.doc]

**Mechanical Strain Stabilizes Reconstituted Collagen Fibrils Against Enzymatic Degradation by Mammalian Collagenase Matrix Metalloproteinase 8 (MMP-8)**

**Brendan P. Flynn^a^, Amit P. Bhole^a^, Nima Saeidi^a^, Melody Liles^a^, Charles A. DiMarzio^a, b^ and Jeffrey W. Ruberti^a,1^**

^a^Mechanical and Industrial Engineering, Northeastern University, Boston, MA 02115; ^b^Electrical and Computer Engineering, Northeastern University, Boston, MA 02115

**Supporting information**

**Protocol bias evaluation**. Because of the small scale of the experiment, differential strain effects on the matrix density, the use of DIC optical imaging/edge detection to assess degradation and the low-mixing digestion protocol it is important to rule out possible bias in the results. Bias could have been introduced by DIC shear axis effects, enzyme concentration gradients, and differences in the network density caused by pipette manipulation. These and other issues have been fully addressed in the SI text.

**Directional edge intensity was examined in addition to overall edge intensity.** Because DIC has a natural shear-axis induced bias, directional edge intensity was examined as well as total edge intensity. A difference in the sensitivity to the edges was found which altered the significance plot (Figure 4). However, the result is similar with loaded fibrils being statistically significantly more stable in the important range of degradation (50% loss of fibrils down to the probable edge intensity threshold of 80% loss of fibrils).

**Internal paired controls remove small solution volume error.** In small reaction volumes, solution inhomogeneities, small pipetting errors and difficulty controlling pH can alter the rate of reaction across samples. Unstretched fibrils, on the periphery of the view field, provide excellent experimental controls for the experimental fibrils stretched between pipettes. Control groups are a maximum distance of 50µm away from experimental groups, and thus experience relatively similar temperature and enzyme concentration, the only difference being the stretch applied to the experimental fibrils.

**Diffusion delays in the chamber were not a significant factor.** The enzyme is introduced into the microchamber by dropping activated MMP-8 solution through an oil layer which could result in enzyme diffusion delays. Multiple ROIs were used to determine if there were significant differences in enzyme arrival across the field of view. For ideal experiments where pipettes drifted less than 10µm and all 10 ROIs were usable (N = 3), T_end_ showed little dependence on ROI location. Degradation times varied between experiments, suggesting variation in time for the enzyme diffusion front to reach the focal plane, but this was not a problem because paired controls, within 50µm of experimental loaded fibrils, were used for each experiment. Degradation patterns indicative of a diffusion front within the field of view were not discernable.

**Comparison of Enzyme activity estimates to data**

Using parameters reported by Gioia et al (Michaelis-Menten K_m_ 5.33e-5M, k_cat_ 4.75s^-1^ ^1^, and reaction-diffusion solutions for enzymatic erosion of insoluble collagen fibrils from Tzafriri et al ^2^ we estimated a total degradation time of 27 min. This estimate, based on an ideal mixing assumption, provides a lower bound because it neglects the time necessary for added enzyme to diffuse to the focal plane and field of view. Based on MMP-8 activity of 1unit/ug, reported by supplier Chondrex, we estimated the same total degradation time of 27 min. This estimate, also assuming ideal mixing and based on activity for collagen molecules in solution, instead of insoluble fibrillar collagen, is also a lower bound. Chondrex recommends dividing activity by a factor, up to 10, to account for reduced substrate availability due to insolubility of fibrils ^3^. Reducing activity by a factor of 10 leads to an expected degradation time of 4.5 hours. This estimate provided an upper bound to actual experimental end times (typically 2-4 hours), because it neglects the difference between complete degradation time (all collagen degraded) and measured degradation time (threshold measureable by our DIC edge intensity routine). That the activities provided by Chondrex, pertaining directly to the MMP-8 used in this investigation, provided accurate upper and lower bounds is to be expected. It appears likely that either the MMP-8 provided by Chondrex had an activity lower than that of the enzyme analyzed by Gioia et al, or the time delay for MMP-8 to diffuse to the focal plane is longer than expected, and the factor of 10 recommended to adjust MMP-8 activity on insoluble fibrillar collagen was unnecessary. Regardless of the explanation, paired controls allow us to extract useful information from the degradation experiments.

**Edge intensity analysis**

Analyses using Mie scattering theory for infinite cylinders to simulate light scattering patterns by uniform diameter collagen networks reveal a linear relationship between gross fibril diameter and integrated edge intensity when using our edge intensity integration routine ^4,5^. While experimental gels comprise a distribution of fibril diameters, the linear relationship between edge intensity and fibril diameter makes possible the comparison of degradation rates between ROIs with different fibril diameter distributions. The edge intensity integration method weights edges based on brightness (Thickness of fibrils), so the calculated edge intensity is truly representative of normalized degradation of the larger fibrils from each ROI.

**Acknowledgements**

Research funded by NIH NIAMS R21 AR053551-01 (PI – Ruberti)

**References**

1. Gioia, M.*, et al.* Modulation of the catalytic activity of neutrophil collagenase MMP-8 on bovine collagen I. Role of the activation cleavage and of the hemopexin-like domain. *J Biol Chem* **277**, 23123-23130 (2002).

2. Tzafriri, A.R., Bercovier, M. & Parnas, H. Reaction diffusion model of the enzymatic erosion of insoluble fibrillar matrices. *Biophysical Journal* **83**, 776-793 (2002).

3. Welgus, H.G., Jeffrey, J.J., Stricklin, G.P., Roswit, W.T. & Eisen, A.Z. Characteristics of the action of human skin fibroblast collagenase on fibrillar collagen. *The Journal of Biological Chemistry* **255**, 6808-6813 (1980).

4. Bohren, C.F. & Huffman, D.R. *Absorption and scattering of light by small particles*, (Wiley, New York, 1983).

5. Bhole, A.P.*, et al.* Mechanical strain enhances survivability of collagen micronetworks in the presence of collagenase: implications for load-bearing matrix growth and stability. *Philos Transact A Math Phys Eng Sci* **367**, 3339-3362 (2009).
